# Supplementary material for: Single pixel imaging at megahertz switching rates via cyclic Hadamard masks
Source: Nat Commun. 2021 Jul 26;12:4516. doi: 10.1038/s41467-021-24850-x (PMC8313532; doi:10.1038/s41467-021-24850-x)
Supplement: Supplementary file 1 — Supplementary Information [file 41467_2021_24850_MOESM1_ESM.pdf]

Single pixel imaging at megahertz switching rates via cyclic Hadamard masks

Supplementary material

Evgeny Hahamovich*, Sagi Monin*, Yoav Hazan and Amir Rosenthal

* Equal contribution

Supplementary note 1: System setup details

A detailed system setup is presented in Supplementary Figure 1. The light emitted by a 625 nm LED (Thorlabs, M625L4) was focused on an area of the coded mask. The spatially coded light at the mask's output was collected by an infinity corrected objective lens (Nikon, N10X-PF). A small mismatch between the stage's center of rotation and center of the circular pattern led to a periodic movement of the coded light in the radial direction, corresponding to a periodic change in the angle of the beam at the infinity space of the objective lens. To compensate for the angular movement of the beam, a piezo-controlled tip/tilt mirror (Physical Instruments, S-335) was used. The control signal to the mirror (Physical Instruments, E727.x) was synchronized with the mask rotation rate and created a periodic change in the mirror angle, compensating for the tilt of the beam. The beam was then directed to a tube lens, which formed a ×10 magnified image of the projected pattern in the image plane. In the image plane, an aperture was positioned in front of the imaged object to block light originating from mask regions outside the relevant coded region. The light passing through the imaged object was collected via a 35 mm convex lens and focused on a single photodetector (Thorlabs, DET 36A-Si). The output signal from the photodetector was amplified by a transimpedance amplifier and captured by a digital oscilloscope (Keysight, DSOX4154A). The mask was rotated at a frequency of up to 10 Hz by an air-bearing rotation stage (Physical Instrument, A-625.025) controlled by a motion controller (Physical Instrument, C891.130300).

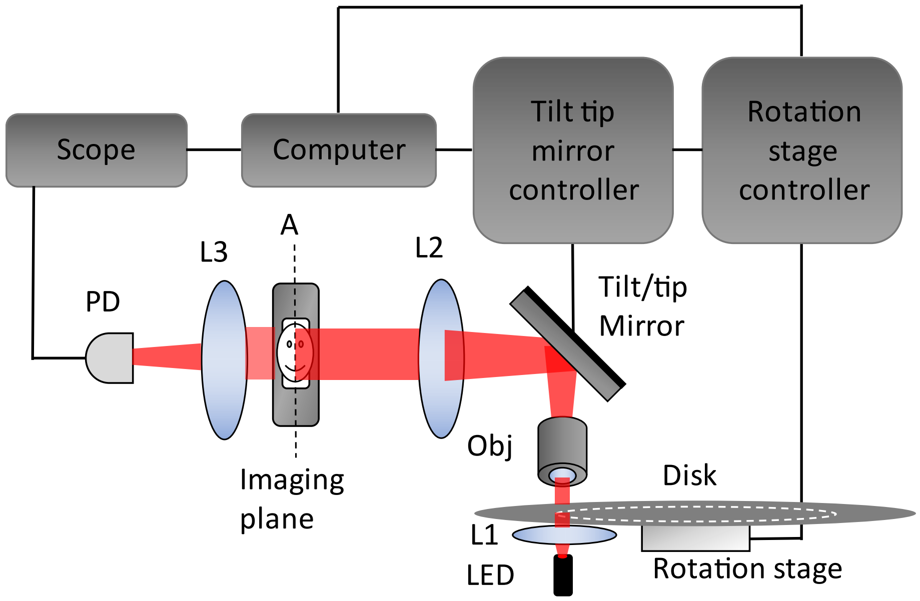

Supplementary Fig. 1 | Detailed system setup. L1 – Collimation lens, L2 – Tube lens, L3 – Focusing lens, Obj – objective lens, A – aperture, PD – photodiode. Both the object and the aperture are placed in the imaging plane. The lines between the different components indicates communication between them.

Supplementary note 2: Tiling geometry

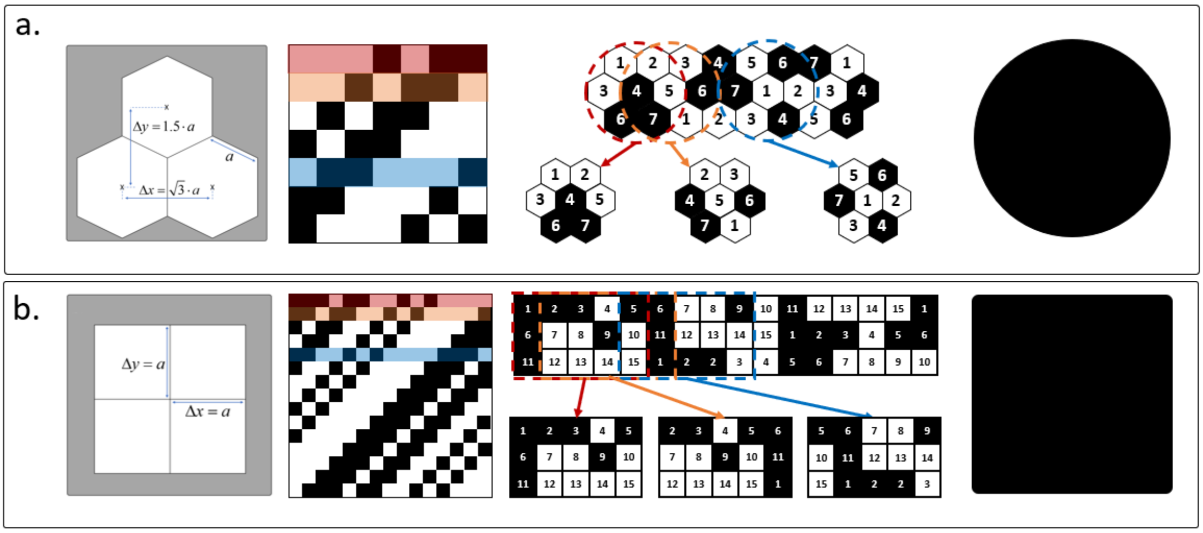

Supplementary Fig. 2 | Pattern geometry. a, from left to right: Pattern geometry for hexagon grid, an example of S-matrix created with Quadratic Residue algorithm with *N*=7 elements, an illustration of the element arrangement on the quartz plate, and the circular geometry of the illumination pattern that was coded to *N* pixels. The different colors correspond to the different coded patterns (pattern 1, 2 and 5). **b,** from left to right: Pattern geometry for square grid, an example of S-matrix created with twin-prime algorithm with *N*=15 (3x5) element, an illustration of the elements arrangement on the quartz plate, and the rectangle geometry of image size of *N*=*P*x*Q* pixels. The different colors correspond to the different coded patterns (pattern 1,2 and 5).

Two types of tiling geometries were used in this work: hexagonal elements combined with circular illumination beam shape (Supplementary Figure 2a) and square elements combined with rectangle illumination beam shape (Supplementary Figure 2b). In both cases, the 1D cyclic codes were transformed into 2D patterns by replicating the 1D code over all the rows of the 2D, where each row was cyclically shifted by several elements with respect to the preceding row. Using this arrangement, a linear translation in the 2D pattern corresponded to cyclic shifts in the 1D code, as illustrated in Supplementary Figure 2. In both tilling geometries, the illumination coming from outside of the required pattern area was blocked by an aperture.

In the following list, we specify the mask parameters used for each of the images and videos produced in our work:

1. Fig. 3a, 3c, and 3d – Circular illumination beam divided to hexagon elements grid with *a*=3 µm, corresponding to a horizontal distance of 5.2 µm and a vertical distance of 4.5 µm between the elements. An objective lens on the optical channel magnified the pixels by 10 during the projection to the image plane. Accordingly, the diameter of the imaged region was 8.7 mm.
2. Fig. 3b – Circular illumination beam divided to hexagon elements grid with *a*=0.85 µm, corresponding to a horizontal distance of 1.47 um and vertical distance of 1.275 um between the elements. An objective lens on the optical channel magnified the pixels by 10 during the projection to the image plane. Accordingly, the diameter of the imaged region was 2.46 mm.
3. Fig. 3e – Rectangle illumination beam divided to square elements grid with *a*=4 µm. Two combined objective lenses (x10 and an inverted x20) on the optical channel decreased the size of the pixels by 2 during the projection to the image plane. Accordingly, the diameter of the imaged region was 0.2 mm.
4. Fig. 4a and the corresponding video recordings – Rectangle illumination beam divided to square elements grid with *a*=4 µm. An objective lens on the optical channel magnified the pixels by 10

during the projection onto the image plane. Accordingly, the diameter of the imaged region was 4 mm.

5. Fig. 4b and 4c and the corresponding video recordings – Circular illumination beam divided to hexagon elements grid with *a*=3 µm, corresponding to a horizontal distance of 5.2 µm and a vertical distance of 4.5 µm between elements. Two combined objective lenses (x10 and an inverted x20) on the optical channel decreased the size of the pixels by 2 during the projection onto the image plane. Accordingly, the diameter of the imaged region was 0.44 mm.

In our analysis, we assumed that all elements in each 2D pattern were of the same size. However, since the patterns were fabricated over a circular path, small differences were obtained between inner and outer diameters of the pattern, leading to minor variations in the element size over the pattern's width. However, as we show in the following, the small difference between the inner and outer diameters of the geometrical ring over which pattern was fabricated led to negligible variation in the elements' width. For example, for the images presented in Fig. 4a in the paper, the aperture size was $404\ \mu m \times 412\ \mu m$, and the pattern was located $57.2\ mm$ away from the center of rotation, depicted in Supplementary Figure 3. Therefore, the difference between the inner and the outer element size was approximately $0.03\ \mu m$, corresponding to a mere 0.75% maximum change in element size. Furthermore, since the critical-dimension uniformity of the photomask was also approximately $0.03\ \mu m$, the geometrical variation in element size falls within the manufacturing-error range.

Supporting calculation:

Angle: $\theta = \arctan\left(\frac{412}{57200}\right) = 0.0072$

Total width for external radius: $57402 \cdot \tan(0.0072) = 413.89\ \mu m$

Total width for internal radius: $56998 \cdot \tan(0.0072) = 410.4\ \mu m$

Total difference is $3.4\ \mu m$, and element difference is $\frac{3.4}{103} = 0.03\ \mu m$

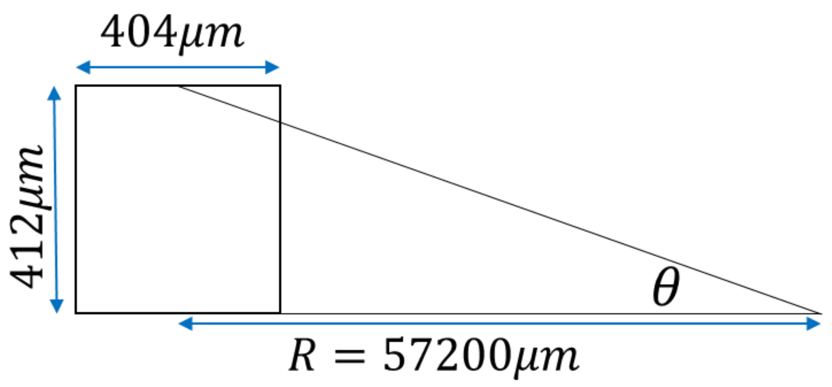

Supplementary Fig. 3 | Pattern variation due to the circular geometry of pattern aperture. Example for Fig.4a of the paper, with pattern distance of $57.2mm$ and element size of $4\mu m$ results in difference of $30nm$ for element size.

Supplementary note 3: Preliminary calibration

Since the center of rotation and the center of the fabricated patterns did not perfectly coincide, rotation led to a harmonic movement of the patterns in the radial direction, which translated to a lateral movement of the illumination pattern on the imaged object. To compensate for this eccentricity and minimize the lateral movement of the illumination pattern, a tip/tilt mirror, positioned in the infinity space of the objective lens (Supplementary Figure 1) was scanned to create a counter lateral movement in the image plane, using the following protocol:

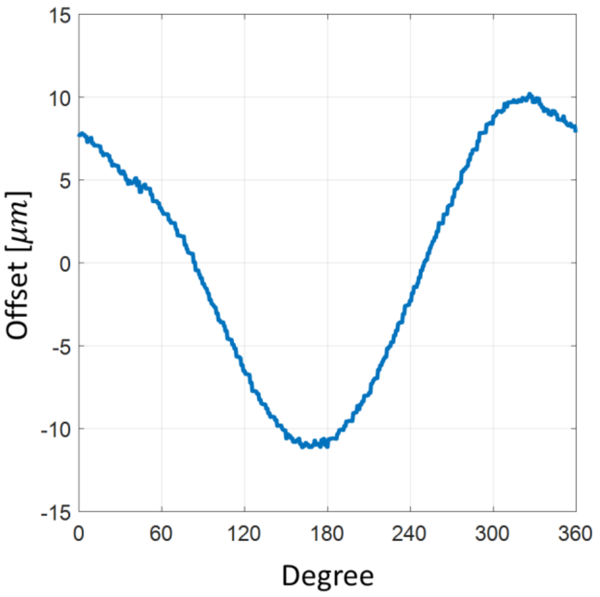

Supplementary Fig. 4 | Eccentricity measurement of quartz plate. Measured eccentricity of the of the system. The plate was rotated through all angles in the range of 0-360 degrees.

1. **Radial-shift measurement:** The imaged object was replaced by a camera (Thorlabs, DCC1545). A calibration pattern of circumference was drawn on the quartz mask and was projected on the camera. The disk was rotated through all the measured angles and the offset of the projected circumference was measured per angle. The results of this eccentricity measurement are presented in Supplementary Figure 4.

2. **Calibration:** Based on the recorded offset, an arbitrary waveform was programed to the tilt/tip mirror controller (Physical Instrument, E727.x).

3. **Real-time compensation:** During the measurements, the mirror was continuously moved based on the injected waveform, compensating for the eccentricity of the system.

An additional effect of the radial movement of the quartz masks is a modulation of the tangential velocity of the projected pattern. Specifically, since the tangential velocity is a product of the approximately constant rotation velocity and the pattern's radius, the harmonic perturbation in the radius is linearly translated into harmonic variation in the tangential velocity and in the rate in which the base functions are switched. To compensate for this effect, the measured offset was used to update the sampling grid during the reconstruction process of the image.

Supplementary note 4: Velocity Tracking Method

Although no drift in the rotation speed was observed in our experiments, it is desirable for our scheme to be robust against such drifts to make it compatible with less accurate stages. To compensate for potential velocity drifts, the tangential velocity should be monitored in real time. In this section we describe a method for velocity monitoring that can be integrated in our imaging setup.

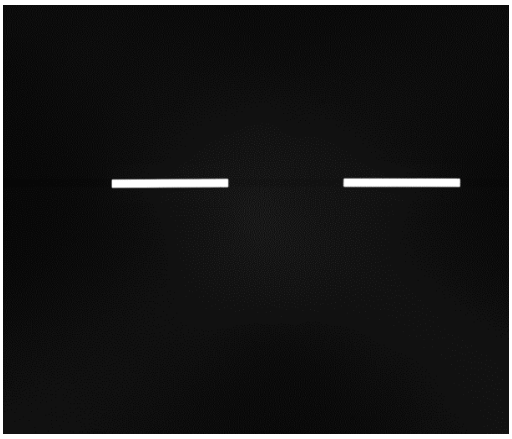

Supplementary Fig. 5 | Segmented lines fabricated on circumference of the mask. The image was captured with camera.

To enable real-time monitoring of the rotation speed, we fabricated a periodic pattern on the photomask, composed of segmented lines with 50% duty-cycle presented (Supplementary Figure 5). A light source was focused onto the pattern with a spot size significantly smaller than the pattern's width. The light transmitted through the segmented lines was measured by a photodiode, leading to a signal that alternated between the two states. Denoting the length of one full cycle by x, and the alternation time between two consecutive cycles with t_i, the velocity was calculated using $v_i = x/t_i$.

This configuration was experimentally examined by measuring the rotation speed of an accelerating stage. Measurement results are shown in Supplementary Figure 6, showing the signal captured by the photodiode (Supplementary Figure 6.a and 6.b.) and the extracted velocity (Supplementary Figure 6.c).

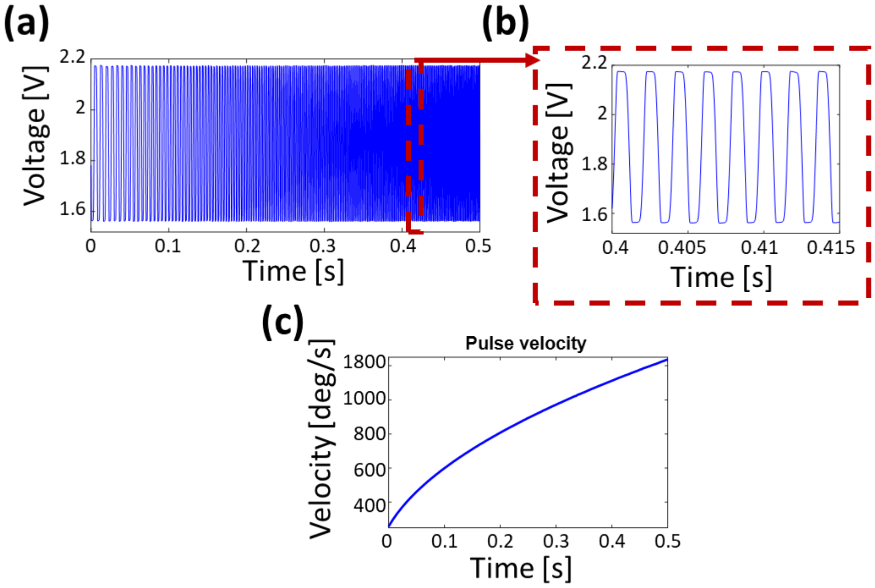

Supplementary Fig. 6 | Velocity measurement using the segmented line pattern. (a) voltage from the photodetector recorded during stage acceleration, (b) zoom on the captured pattern and (c) the recovered velocity of the stage as a function of time.

The technique described above may be used to monitor the rotation speed of the photomask during the imaging session by adding a second photodetector to capture the transmission pattern from the periodic pattern. Then, random drifts in velocity may be corrected in the post processing of the data, using the same procedure applied for the deterministic velocity changes in Section 4 of the Supplementary Information.

Supplementary note 5: Image reconstruction and sampling matrix procedure

In our work we used $\mathbf{S}$-matrix codes as the sampling matrix. The $\mathbf{S}$-matrix is a well-known code based on Hadamard codes[1]. In contrast to Hadamard codes, which are based on binary code of $\pm 1'$, $\mathbf{S}$-matrix codes are based on $0'$s and $1'$s. $\mathbf{S}$-matrices have Paley construction, where the sampling matrix is cyclic construction, where each row of the matrix is cyclic left shift of the previous row.

The reconstruction can be generally performed via a matrix multiplication of $\hat{\mathbf{x}} = \mathbf{S}^{-1}\mathbf{y}$, where $\hat{\mathbf{x}}$ is a row stack vector representation of the recovered image, $\mathbf{S}^{-1}$ is the inverse of the projection matrix and $\mathbf{y}$ is the measurement vector. The S-matrix has a closed-form solution for inverse matrix calculation $\mathbf{S}^{-1} = \frac{2}{N+1}(2\mathbf{S}^T - \mathbf{J})$, where $\mathbf{J}$ is a matrix of 1's and N is the size of the matrix. However, performing the reconstruction with a matrix-vector multiplication leads to computational complexity of $O(N^2)$ and memory requirements of of $O(N^2)$, which may limit the implementation for large images. For example, in our work the matrix occupied ~5 Gb of memory for $N = 25{,}111$ when double-class precision.

To accelerate the reconstruction, we propose an alternative algorithm that is based on Fast Fourier Transform (FFT). Using the property of cyclic sampling, the sampling equation $\mathbf{y} = \mathbf{S}\mathbf{x}$, can be expressed as $\mathbf{y} = \mathbf{s_1} \circledast \mathbf{x}$, where $\mathbf{s_1}$ is the first row of the sampling matrix, and $\circledast$ is the circular convolution operator. By circular convolution theorem[2] we can express the sampling equation with FFT: $F\{\mathbf{y}\} = F\{\mathbf{S_1}\} \cdot F\{\mathbf{x}\}$, where $F\{\}$ denotes the FFT operator, and the image can be reconstructed by $\hat{\mathbf{x}} = F^{-1}\{F\{\mathbf{y}\}/F\{\mathbf{S_1}\}\}$. Reconstruction with FFT is of computational complexity of $O(N\log N)$ and memory requirements of $O(N)$, accelerating reconstruction time greatly and reducing memory requirements, thus facilitating the reconstruction of large images. In this work, the reconstruction of images with $N = 25{,}111$ was performed in 4 ms on a CPU without any parallel computations and required a memory of 0.4 Mb with complex double-class precision for storing $F\{\mathbf{S_1}\}$.

Supplementary note 6: Comparison to a reference camera image

To demonstrate the capability of our scheme for imaging gray-scale images, we tested it on an old photographic film containing an image of flowers. The resulting SPI reconstruction, presented in Fig. 3d of the main paper, is compared in this section to an image of the film taken with a scientific camera (Thorlabs, DCC1545). For a fair comparison between the images we resized the camera image from the original 1280 x 1024 pixels to the resolution of the SPI image. The comparison presented in Supplementary Figure 7, shows a very good visual agreement between the two images.

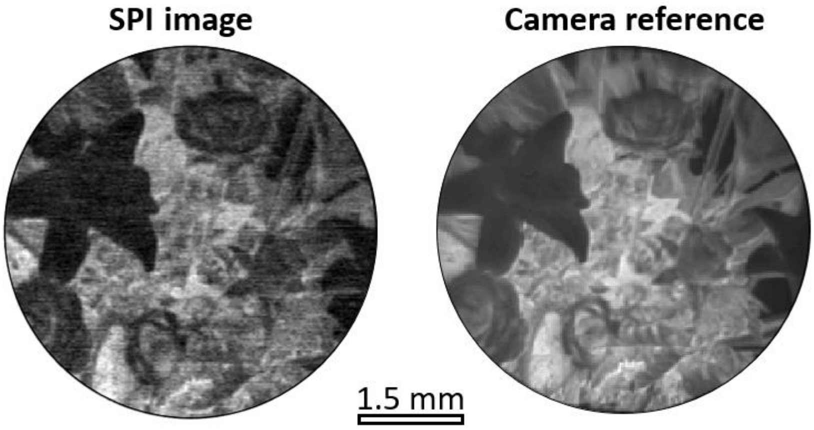

Supplementary Fig. 7 | Gray level image of a flowers slide. An image of an old photographic slide with gray levels captured with our SPI system (left) is compared to a reference image (right) taken with a camera.

Supplementary note 7: The effect of averaging sub-pixel-shifted images

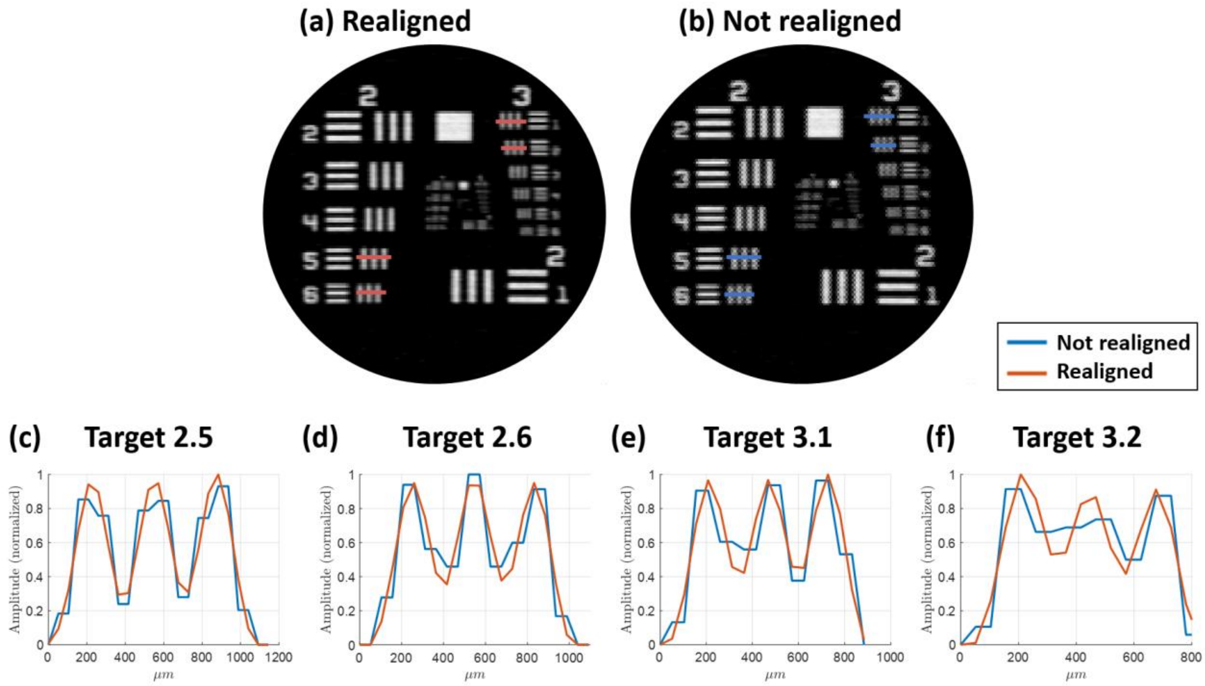

Supplementary Fig. 8 | Comparison of image reconstruction from over-sampled data with and without image realignment. M =5 slightly shifted experimental reconstructions of a resolution target were produced and summed to produce the final reconstruction (a) with and (b) without performing sub-pixel realignment before summation. (c)-(f) a comparison between the 1D slices of resolution targets 2.5, 2.6, 3.1 and 3.2, reconstruction with (red) and without (blue) realignment.

In this section, we demonstrate the benefit of using sub-pixel shifts in the image reconstruction, as discussed in Fig. 2 of the main paper. During the measurement, the analog signal is sampled at a high sample rate, with a temporal resolution significantly above the switching time between two neighboring patterns, T. For an over-sampling factor of M, we obtain M distinct reconstructions, which are shifted with respect to one another by Δ/M, where Δ is the pixel width; all M images are realigned and averaged to obtain the final reconstruction.

As demonstrated in the following examples, averaging the images after sub-pixel realignment can produce images with higher contrast in comparison to direct approach in which realignment is not used and better SNR in comparison to a single sub-pixel reconstruction. In the first example, we consider an alternative reconstruction strategy in which the images are averaged without realignment. Supplementary Figure 8 shows the resulting reconstructions of the resolution target shown in Fig. 3.a with (Supplementary Figure 8a) and without (Supplementary Figure 8b) realignment and compares 1D slices taken over various positions on the targets (Supplementary Figure 8c-8f). As can be seen in the reconstructions of Targets 3.1 and 3.2, realigning the images enhances the contrast of fine details in the image.

In the second example, we tested the SNR gain achieved by averaging the M images over using only a single image on the example of Fig. 3.b of the manuscript, which was captured with the maximum imaging rate of our system, 2.4 megapixel/sec. Generally, for sufficiently high imaging rates the main

noise source in the reconstructions is noise in the photodetector output, whose magnitude is proportional to the square root of the imaging duration. Under this noise model, reconstructions performed by averaging M realigned images will lead to a $\sqrt{M}$ SNR gain in comparison to a single image of M reconstructions. Supplementary Figure 9 shows the SNR gain as a function of M in comparison to the theoretical prediction and demonstrates a maximum measured SNR gain of 2.6 for averaging of 33 sub-pixel shifts. The noise was evaluated using the dark regions of the image. Since the reconstruction was not ideal, and even dark regions included some structure, we first performed background subtraction by calculating the difference between two subsequent frames and then evaluating the standard deviation over the difference image at the dark regions. The signal amplitude was determined by calculating the average value over the white image regions. The saturation in the measured SNR gain at high values of M, in contrast to the theoretical prediction, may be explained by structural noise in the reconstructions due to minute inaccuracies in the motion of the photomask, which are correlated between the M images.

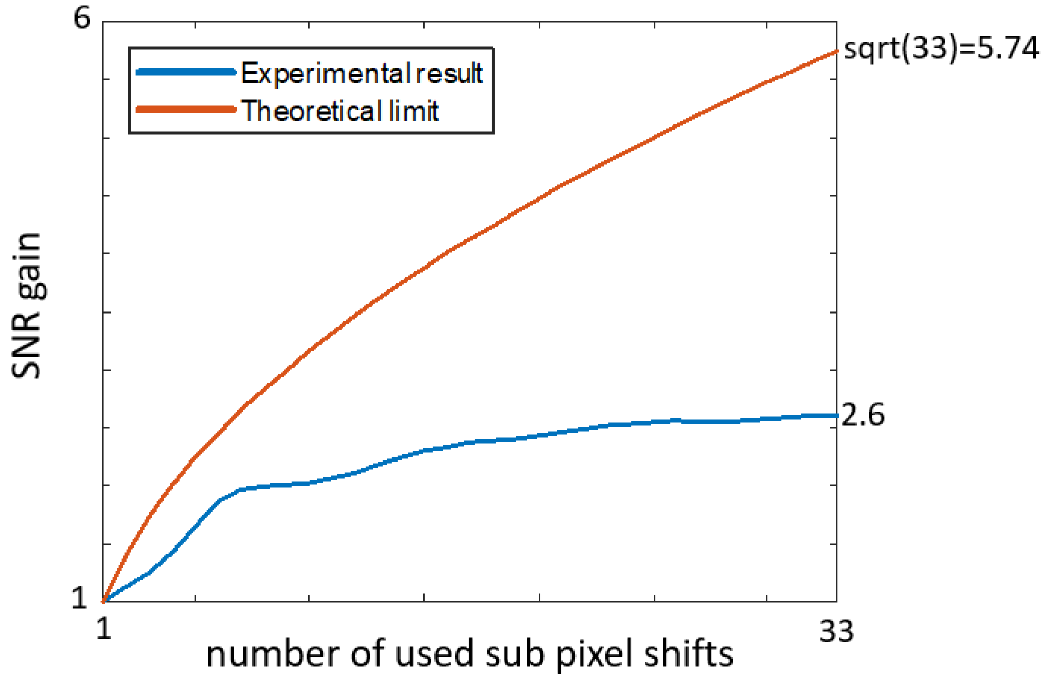

Supplementary Fig. 9 | SNR gain achieved by the sub-pixel shift. SNR gain of averaging M realigned images in comparison to a single under-sampled reconstruction for the image presented in Fig. 3b.

Supplementary note 8: Sampling matrix formation procedure

The S-matrix codes were generated by the Quadratic Residue algorithm for the hexagon patterns (Supplementary Figure 2.a) and by the Twin Prime algorithm for square patterns (Supplementary Figure 2.b)[1]. Detailed construction algorithms are:

Algorithm 1: Quadratic Residue Construction of S-matrix		
Input	:	n - number of elements in matrix. The number must be a prime number of the form $4m+3$, where m is an integer
Step 1	:	Create vector: $a=[1,4,9,...,((n-1)/2)^2]$
Step 2	:	Calculate remainder of a by n, $b=rem(a,n)$
Step 3	:	Create a vector of zeros $s(1:n)=0$.
Step 4	:	Add +1 to vector s in b indexes, $s[b]=1$
Step 5	:	For $i=0:n-1$<br>Cyclic shift vector s to the left by i, $\tilde{s}=shift(s,i)$<br>Insert vector to i^{th} row of matrix, $\mathbf{S}[i,:]\leftarrow\tilde{s}$<br>end

Algorithm 2: Twin-prime Construction of S-matrix		
Input	:	p - where p and $q=p+2$ are both prime number and $n=p\times q$ - number of elements in matrix
Step 1	:	Create vector: $a=0:n-1$
Step 2	:	Calculate remainder of a by p, $b_f=rem(a,p)$
Step 3	:	Create vector: $c_f=[1,4,9,...,((p-1)/2)^2]$
Step 4	:	Calculate remainder of c_f by p, $d_f=rem(c_f,p)$
Step 5	:	Create a vector of -1, $f(1:n)=-1$.
Step 6	:	Add +1 to vector f in b_f indexes, $s[b_f]=0$
Step 7	:	For $i=0:q-1$<br>Add +2 to vector f in $d_f+i\times p$ indexes, $s[d_f+i\times p]=1$<br>end
Step 8	:	Calculate remainder of a by q, $b_g=rem(a,q)$
Step 9	:	Create vector: $c_g=[1,4,9,...,((q-1)/2)^2]$
Step10	:	Calculate remainder of c_g by q, $d_g=rem(c_g,p)$
Step11	:	Create a vector of -1, $g(1:n)=-1$.
Step12	:	Add +1 to vector g in b_g indexes, $s[b_g]=0$
Step13	:	For $i=0:p-1$<br>Add +2 to vector g in $d_g+i\times p$ indexes, $s[d_g+i\times p]=1$<br>end
Step14	:	Create a vector of ones $s(1:n)=1$.
Step15	:	Set 0 to vector s in indexes where $f==g$, $s[f==g]=0$
Step16	:	Set 0 to vector s in indexes $[0,q,2q,...,(p-1)q]$, $s[0,q,2q,...,(p-1)q]=0$
Step17	:	For $i=0:n-1$<br>Cyclic shift vector s to the left by i, $\tilde{s}=shift(s,i)$<br>Insert vector to i^{th} row of matrix, $\mathbf{S}[i,:]\leftarrow\tilde{s}$<br>end

Supplementary note 9: Compressed sensing

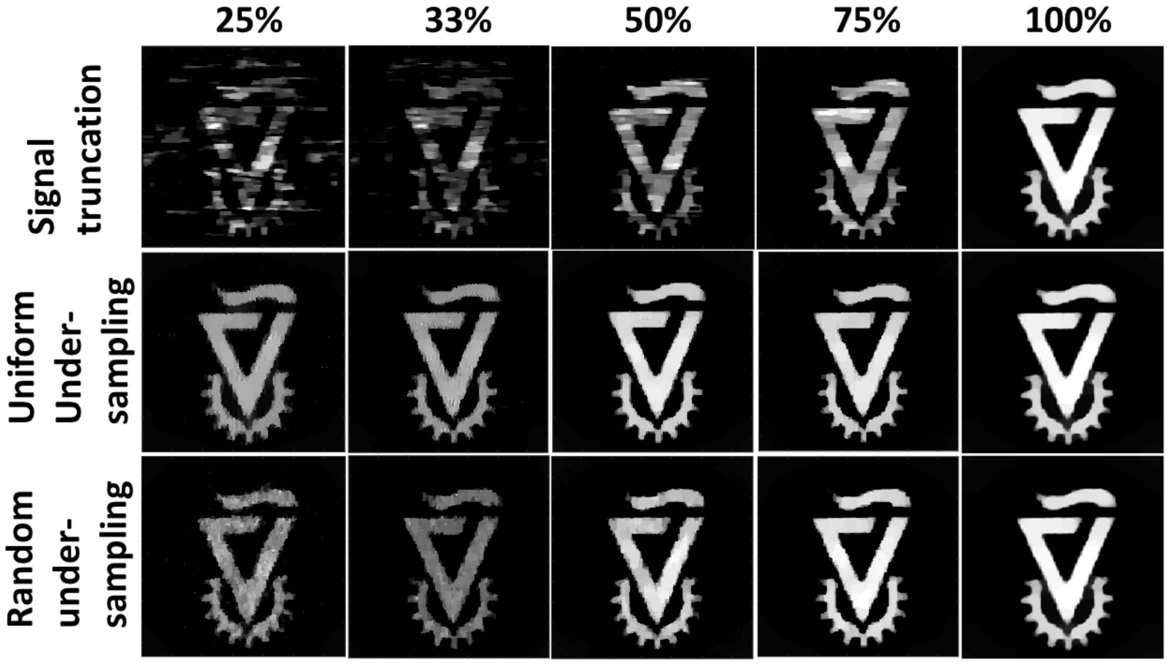

Supplementary Fig. 10 | Compressed sensing image restoration based on partial data. The Technion logo image is recovered from partial samples with different sampling procedures.

SSIM	25%	33%	50%	75%
Signal truncation	0.6	0.66	0.82	0.88
Uniform Under-sampling	0.79	0.89	0.93	0.96
Random under-sampling	0.81	0.85	0.9	0.92

Supplementary Table 1 | SSIM comparison of the CS reconstructions. Comparison was conducted between CS results and a full base reconstruction with 100% of the measurements.

PSNR	25%	33%	50%	75%
Signal truncation	12.35	12.44	15.55	18.3
Uniform Under-sampling	18.55	18.59	26.46	27.41
Random under-sampling	16.68	14.98	22.77	25.76

Supplementary Table 2 | PSNR comparison of the CS reconstructions. Comparison was conducted between CS results and a full base reconstruction with 100% of the measurements.

While the results presented in the paper use full base acquisition and reconstruction, it is possible to reduce the required number of measurements by algorithmic reconstruction optimization via compressed sensing. We demonstrate this by performing image reconstruction from under sampled datasets with 75%, 50%, 33% and 25% of the samples. We show a comparison of three under-sampling methods:

1) **Signal truncation:** The signal is truncated, leading to a reduced dataset in which samples are consecutive.
2) **Uniform under-sampling:** We take every n^{th} sample of the original dataset, where $n = 2,3,4$, to form the reduced dataset. For example, for 75% we omit every 4th measurement.
3) **Random under-sampling:** The samples of the reduced dataset are randomly chosen from the full dataset.

For all cases, the reconstruction was performed by a TVAL3 algorithm[3], solving a compressed sensing problem with total variation regularization. This algorithm was proposed for single-pixel imaging[4] and demonstrated in different SPI scenarios[5–7]. Identical hyper-parameters were used for all the compared reconstructions.

The reconstruction results are presented in Supplementary Figure 10 for the Technion logo. To quantify the reconstruction quality, the SSIM and PSNR values were calculated between the CS reconstructions and the full-data reconstruction, using the following equations:

For two images, $\mathbf{I}$ and $\mathbf{J}$, structural similarity (SSIM) is calculated by the following equation:

$$SSIM = \frac{(2\mu_I\mu_J + c_1)(2\sigma_{IJ} + c_2)}{(\mu_I^2 + \mu_J^2 + c_1)(\sigma_I^2 + \sigma_J^2 + c_2)},$$

where μ_I and μ_J are averages of the two compared images, σ_I^2 and σ_J^2 are the variances of the images, σ_{IJ} is the covariance, and c_1 and c_2 are two variables calculated from the dynamic range of the images.

For two images, $\mathbf{I}$ and $\mathbf{J}$ of sizes $[X, Y]$, peak signal-to-noise ratio (PSNR) is calculated by the following equation:

$$PSNR = 10\log_{10}\left(\frac{MAX_I^2}{MSE}\right) = 10\log_{10}\left(\frac{MAX_I^2}{\frac{1}{XY}\sum_{x=0}^{X-1}\sum_{y=0}^{Y-1}|I(x,y) - J(x,y)|^2}\right),$$

where MAX_I is the maximum values over the image I.

The SSIM and PSNR values are summarized in Supplementary Tables 1 and 2. The figure and tables clearly show that uniform and random under-sampling led to better reconstructions than signal truncation. Nonetheless, even in the case of signal truncation, the Technion logo was clearly visible with 50% of the measurement data. Accordingly, it should be possible to increase the imaging rate by 2 while acquiring only half of the base and reconstructing the image with the described compressive sampling algorithms, achieving imaging rate of up to 4.8 mega pixels per second.

Supplementary note 10: Video recording details

Three videos captured by our system are attached as Supplementary Videos 1-3. Several frames from each video are presented in Fig. 4 of the manuscript. Each frame followed the same reconstruction procedure as the individual images, as described in Supplementary note 5.

Detailed configuration per video recording are listed below:

1. Resolution target motion (Fig.4.a in the manuscript, Supplementary Video 1): Total of 142 consecutive frames, captured at a frame rate of 72 fps with 10,403 pixels per frame. The video captures a vertical scan of a standard resolution target. A rectangle illumination beam divided to square elements grid (Supplementary Figure 2.b.) was used, with x10 optical magnification of the object, leading to a pixel size of 40 um (4 um x 10).

2. Worm motion (Fig. 4b and 4c in the manuscript, Supplementary Video 2 and 3): Total of 31 consecutive frames captured at a frame rate of 10 fps with 25,111 pixels per frame. The videos captured the in vivo motion of *C. elegans* worms. A circular illumination beam divided to hexagon elements grid (Supplementary Figure 2a) was used, with x0.5 magnification of the object, leading to a pixel size of 2.6 µm.

Supplementary References

1. Harwit, M. *Hadamard transform optics*. (Elsevier, 1979). doi:10.1016/B978-0-12-330050-8.50001-9
2. Yin, W., Morgan, S., Yang, J. & Zhang, Y. Practical compressive sensing with Toeplitz and circulant matrices. *Vis. Commun. Image Process. 2010* **7744**, 77440K (2010).
3. Li, C., Yin, W., Jiang, H. & Zhang, Y. An efficient augmented Lagrangian method with applications to total variation minimization. *Comput. Optim. Appl.* **56**, 507–530 (2013).
4. Li, C. *An Efficient Algorithm For Total Variation Regularization with Applications to the Single Pixel Camera and Compressive Sensing*. (2009).
5. Howland, G. A., Lum, D. J., Ware, M. R. & Howell, J. C. Photon counting compressive depth mapping. *Opt. Express* **21**, 23822 (2013).
6. Yang, J. & Zhang, Y. Alternating direction algorithms for ℓ1-problems in compressive sensing. *SIAM J. Sci. Comput.* **33**, 250–278 (2011).
7. Howland, G. A., Lum, D. J. & Howell, J. C. Compressive wavefront sensing with weak values. *Opt. Express* **22**, 18870 (2014).
