# Supplementary material for: Single pixel imaging at megahertz switching rates via cyclic Hadamard masks
Source: Nat Commun. 2021 Jul 26;12:4516. doi: 10.1038/s41467-021-24850-x (PMC8313532; doi:10.1038/s41467-021-24850-x)
Supplement: Supplementary file 3 — Description of Additional Supplementary Files [file 41467_2021_24850_MOESM3_ESM.docx]

**Description of Additional Supplementary Files**

The work includes three supplementary videos showing the motion of the different objects as presented in Fig. 4 of the main manuscript. The legends for the supplementary videos are:

- **Supplementary Movie 1**

This is a video of a vertically shifted resolution target. The legends are:


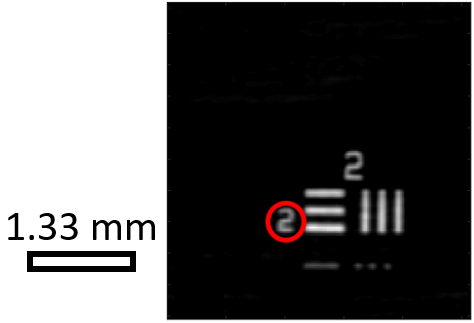


- **Supplementary Movie 2**

This is a video of a live, moving C. elegans worm. The legends are:


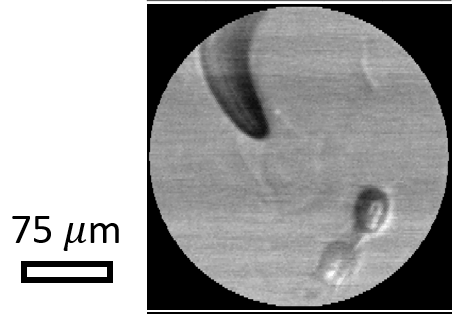


- **Supplementary Movie 3**

This is a video of a live, moving C. elegans worm. The legends are:


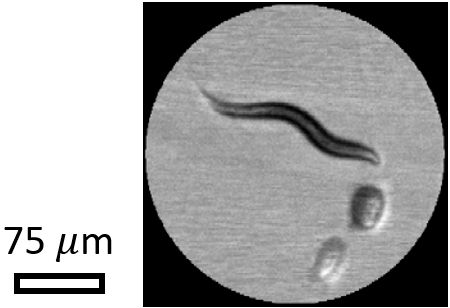


Additional details on the used pixel sizes per movie are found in Supplementary note 10.
