# Supplementary figures and images for: Single pixel imaging at megahertz switching rates via cyclic Hadamard masks
Source: Nat Commun. 2021 Jul 26;12:4516. doi: 10.1038/s41467-021-24850-x (PMC8313532; doi:10.1038/s41467-021-24850-x)

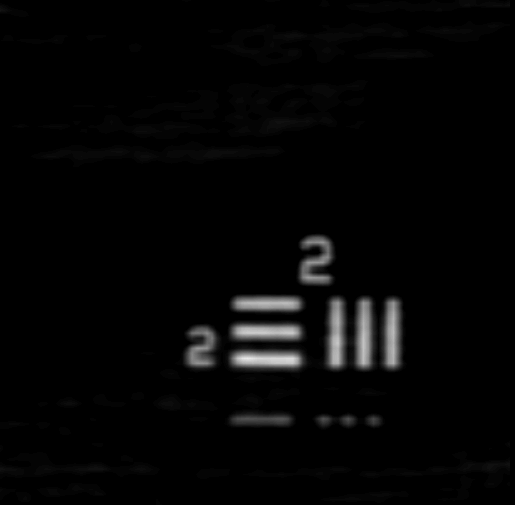

Supplement: Supplementary file 4 — Supplementary Movie 1 [file 41467_2021_24850_MOESM4_ESM.gif]

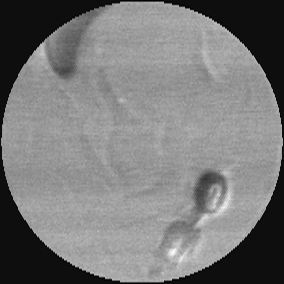

Supplement: Supplementary file 5 — Supplementary Movie 2 [file 41467_2021_24850_MOESM5_ESM.gif]

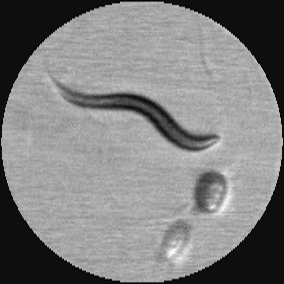

Supplement: Supplementary file 6 — Supplementary Movie 3 [file 41467_2021_24850_MOESM6_ESM.gif]
